# Long-Term Disease Stability and Radiological Progression in Low-Grade Glioma: A Retrospective MRI-Based Cohort Study of Post-surgical and Observation Groups

**DOI:** 10.7759/cureus.117623

**Published:** 2026-10-08

**Authors:** Fariha Ejaz, Memoona Alam, Sahar Aslam, Aasia Jehangir, Syeda Fareesa H Zaidi, Ammara Manzoor, Talha Laique

**Affiliations:** 1 Department of Diagnostic Radiology, Islamabad Diagnostic Centre, Lahore, PAK; 2 Department of Radiology, Chugtai Lab, Lahore, PAK; 3 Department of Radiology, Ali Fatima Hospital, Lahore, PAK; 4 Department of Radiology, Social Security Teaching Hospital, Lahore, PAK; 5 Department of Physiotherapy, Jinnah Hospital, Lahore, PAK; 6 Department of Rehabilitation, Medical Health Services Clinic, Islamabad, PAK; 7 Department of Pharmacology, Lahore Medical and Dental College, Lahore, PAK

**Keywords:** disease progression, low-grade glioma, mri surveillance, progression-free outcome, subtotal resection, tumour volume

## Abstract

Background: Low-grade gliomas (LGGs) are generally known as primary brain tumors that grow over time because of the residual tumor after intervention. A series of MRIs is used to see any radiological progression over a course of time.

Aim: This study aimed to make a comparison between the radiological progression and disease stability for patients with subtotal resection and for those that were managed through observation.

Methods. A total of 94 patients with LGGs who underwent contrast-enhanced MRI of the brain and had their follow-up between July 2021 and July 2026 documented were retrospectively reviewed. The volume of the tumor was recorded before and after the intervention. The patient was considered to have progressed only if the tumor had increased; however, it was considered stable if there was no increase observed or the tumor had been reduced. Fisher exact test was used to compare the groups.

Results. The group (mean age = 35.3 years; range, 8-70 years) consisted of patients who underwent subtotal resection (n=59, 62.8%), observation (n=33, 35.1%), and total resection (n=2, 2.1%). Of the 59 patients, 41 (69.5%) had stable disease after subtotal resection while 18 (30.5%) progressed. In the observation group, 28 out of 32 assessable patients (87.5%) were stable, and four (12.5%) progressed. Progression was more frequently observed after subtotal resection than observation (30.5% vs. 12.5%; OR 3.07, 95% CI 0.94-10.05; p=0.073), although the difference was not statistically significant. Patients who had a complete resection also remained stable.

Conclusion: In this MRI-based retrospective cohort of patients with LGG, most patients remained radiologically stable during follow-up. Progression was more frequent after subtotal resection than observation.

## Introduction

Low-grade gliomas (LGGs) are a heterogeneous group of primary central nervous system tumors characterized by relatively slow growth and a prolonged clinical course. According to the World Health Organization (WHO) classification, conventional grade 1-2 gliomas, with inclusion based on the available histopathological diagnosis, have molecular confirmation that was not mandatory, thus encompassing distinct molecular entities with variable biological behavior and prognosis [1]. In contemporary classification, molecular alterations such as isocitrate dehydrogenase (IDH) mutation and 1p/19q codeletion are important determinants of tumor classification, prognosis, and clinical course [2]. 

Although LGGs generally have a more indolent course than high-grade gliomas, many eventually demonstrate radiological or clinical progression and may undergo malignant transformation [3]. Therefore, management aims to maximize disease control while preserving neurological function and quality of life. Surgical resection remains a central component of treatment, and the extent of resection is an important modifiable factor associated with progression-free and overall survival [4]. However, complete tumor removal may not be feasible when lesions involve or are adjacent to eloquent cortical or subcortical structures, resulting in residual tumor after subtotal resection [5]. Conversely, selected patients with small, asymptomatic, or surgically inaccessible lesions may be managed initially with observation and serial imaging rather than immediate intervention [6].

Longitudinal radiological surveillance is therefore essential for evaluating tumor behavior and guiding subsequent management. Tumor volumetry was performed using the contrast-enhanced T1-weighted MRI sequence, with tumor volume assessed based on the identifiable lesion on post-contrast images; this approach was used consistently across available follow-up examinations [7, 8]. Standardized response assessment frameworks, including the Response Assessment in Neuro-Oncology (RANO) criteria, facilitate objective evaluation of changes in tumor burden [8].

Despite the established role of extent of resection, the longitudinal behavior of residual tumors following subtotal resection compared with initially observed lesions remains clinically relevant. This retrospective cohort study, therefore, evaluated radiological disease progression and changes in tumor volume among patients with LGG managed with subtotal resection or observation, using documented baseline and follow-up contrast-enhanced MRI findings. The findings may contribute to understanding tumor-volume trajectories and support individualized decisions regarding surveillance and subsequent therapeutic management [9].

## Materials and methods

This was a single-center retrospective cohort study conducted at Shaukat Khanum Memorial Cancer Hospital and Research Centre, Lahore, Pakistan. Patients were identified from the institutional MRI/clinical records using the Current Procedural Terminology (CPT®) code 70552 [10] and were included if they had a documented diagnosis of LGG, available baseline and follow-up contrast-enhanced brain MRI between July 2021 and July 2026, and sufficient imaging documentation for assessment of tumor progression. Patients with incomplete or non-interpretable imaging, unavailable follow-up data, or diagnoses inconsistent with LGG were excluded, while pathological confirmation was not mandatory because the study relied on the documented clinical/radiological diagnosis in the retrospective records. A total of 94 patients met the predefined eligibility criteria and constituted the final analysis cohort. Patients were categorized retrospectively according to the therapeutic strategy received: subtotal resection, observation (no treatment/no surgery), or total resection. Extent of resection was classified from operative/surgical records as subtotal resection when residual tumor remained and total resection when the surgeon documented complete tumor removal. Postoperative MRI and a quantitative extent-of-resection threshold were not used for group classification. IDH mutation and 1p/19q codeletion status were not routinely available in this retrospective cohort; therefore, molecular data were missing for all patients (100%), precluding molecular subgroup analysis.

Contrast-enhanced MRI was performed for all patients, with tumor volume measured on the available baseline and follow-up scans. For surgically treated patients, the preoperative MRI was considered the primary baseline, representing the pretreatment tumor burden, and the most recent available postoperative MRI was used to assess overall radiological change following treatment. For patients managed with observation, the earliest available MRI was considered the baseline, and the most recent follow-up MRI was used for assessment. Thus, the primary analysis evaluated overall change in tumor volume from the pretreatment/initial observation state to the most recent available follow-up and was not intended to represent growth of residual postoperative disease alone.

Tumor volume (mL) was measured by manual segmentation of the visible lesion on contrast-enhanced T1-weighted MRI, with lesion boundaries delineated slice-by-slice and summed to obtain the total volume. Measurements were performed by a single experienced radiologist using the institutionally available MRI analysis software; no automated or semi-automated segmentation or inter-reader assessment was performed.

Radiological progression for the primary analysis was defined as a ≥25% increase in tumor volume from the corresponding baseline to reduce the likelihood of classifying minor segmentation or measurement variability as true progression. Stable disease was defined as a change of <25% in either direction, whereas a ≥25% decrease was classified as radiological regression, ensuring mutually exclusive outcome categories.

For patients who underwent subtotal resection and had measurable postoperative residual tumor, a separate secondary analysis was performed to evaluate the subsequent behavior of the residual lesion. The first available postoperative MRI demonstrating residual tumor was designated the postoperative residual baseline. The residual tumor volume on the most recent available follow-up MRI was compared with this postoperative residual baseline. A ≥25% increase was classified as progression of residual disease, a change of <25% in either direction as stable residual disease, and a ≥25% decrease as regression. This secondary analysis was considered separately from the primary preoperative-to-follow-up analysis and was intended specifically to distinguish subsequent growth of residual postoperative tumor from the overall change in tumor burden after surgery [6,8].

Statistical analysis was performed using IBM SPSS Statistics, version 26.0 (IBM Corp., Armonk, NY, USA). Continuous variables were assessed for distribution and summarized as mean ± standard deviation (SD) for normally distributed data or median with interquartile range (IQR) for non-normally distributed data. Categorical variables were presented as frequencies and percentages. Categorical variables were compared using the chi-square test or Fisher’s exact test, as appropriate, with Fisher’s exact test specifically used for progression between the subtotal-resection and observation groups. Continuous tumor-volume data were assessed for distribution and compared between groups using the independent-samples t-test for normally distributed variables or the Mann-Whitney U test for non-normally distributed variables; within-patient baseline-to-follow-up changes were assessed using the paired t-test or Wilcoxon signed-rank test, as appropriate. All statistical tests were two-sided, and a p-value <0.05 was considered statistically significant.

## Results

The group (mean age = 35.3 years; range, eight to 70 years) consisted of patients who underwent subtotal resection (n=59, 62.8%), observation (n=33, 35.1%), and total resection (n=2, 2.1%). A greater baseline (pre-treatment) tumor volume was seen in subtotal resection patients (median 62 mL, IQR 31-120) than in the observation group (median 48 mL, IQR 20-103), whereas greater or symptomatic lesions were selected for surgery (Table 1).

**Table 1:** Tumor volume characteristics and radiological changes according to management strategy Volume change is the median of post- minus pre-treatment volume.

Characteristic	Subtotal resection	Observation	Total resection	Overall
Patients, n (%)	59 (62.8%)	33 (35.1%)	2 (2.1%)	94 (100%)
Age, mean (years)	34.8	36.8	26	35.3
Age range (years)	—	—	—	8–70
Pre-treatment volume, median (mL)	62	48	108	—
Pre-treatment volume, IQR (mL)	31–120	20–103	—	—
Post-treatment volume, median (mL)	62	45	55	—
Median volume change (mL)	−2	0	−3	—

On follow-up, 41 of 59 (69.5%) patients in the subtotal resection group had stable disease, whereas 18 (30.5%) demonstrated radiological progression (Table 2). In the observation group, outcome data were available for 32 of 33 patients; 28 (87.5%) remained stable, and four (12.5%) demonstrated radiological progression. For one patient in the observation group, progression status could not be determined because the relevant follow-up imaging/documentation was unavailable. This patient was, therefore, excluded from the calculation of the proportion of patients with progression and was not classified as either stable or progressive. Both patients who underwent total resection remained stable during follow-up. Table 2 summarizes the observed stability and progression within each management group.

**Table 2:** Radiological outcome by management strategy *One observation patient with undocumented progression status was excluded, leaving 32 assessable; N: accessible patients

Management strategy	N	Stable disease, n (%)	Progression, n (%)
Subtotal resection	59	41 (69.5)	18 (30.5)
Observation (no treatment)	32*	28 (87.5)	4 (12.5)
Total resection	2	2 (100)	0 (0)

Table 3 shows that tumor progression occurred in 18 of 59 patients, 18 (30.5%) in the subtotal resection group compared with four of 32 patients, 4 (12.5%) in the observation group. Progression was almost 2.4 times more common after subtotal resection than observation (odds ratio 3.07); however, this difference was not statistically significant on two-sided Fisher’s exact test (p = 0.073). Table 3 presents the direct comparison of progression rates between the subtotal resection and observation groups, including the odds ratio and Fisher’s exact test.

**Table 3:** Side-by-side comparison of outcome by management strategy *Fisher exact test; insignificant = p-value >0.05; Progression was more frequent after subtotal resection (18, 30.5%) than with observation (4, 12.5%).

Management strategy	Total assessable cohort (n)	Stable disease, n (%)	Disease progression, n (%)	P-value
Subtotal resection	59	41 (69.5%)	18 (30.5%)	0.073
No treatment (observation)	32	28 (87.5%)	4 (12.5%)	

Disease progression occurred more frequently in the subtotal resection group than in the observation group (18/59 (30.5%) vs. 4/32 (12.5%)). The odds of progression were approximately threefold higher in the subtotal resection group (OR 3.07, 95% CI 0.94-10.05); however, this difference did not reach statistical significance on the two-sided Fisher’s exact test (p=0.073) (Table 4). Tumor volume decreased after subtotal resection (median change, −2.0 mL; mean change, −12.5 mL), whereas the observation group showed minimal overall change (median change, 0 mL; mean change, +1.6 mL). These tumor volume changes were presented descriptively. The p-value of 0.073 referred specifically to the comparison of disease progression rates and did not represent an inferential comparison of continuous tumor volume change.

**Table 4:** Tumor volume trajectories and disease progression by management strategy † p=0.073 represents the two-sided Fisher’s exact test comparing the proportion of patients with disease progression between the subtotal-resection and observation groups. No inferential p-value was calculated for the descriptive tumor-volume changes; Negative values indicate volume reduction; positive values indicate increase.

Outcomes	Subtotal resection (n=59)	Observation (n=32)	p-value
Disease progression, n (%)	18 (30.5%)	4 (12.5%)	OR 3.07 (95% CI 0.94–10.05); p=0.073†
Median change in tumor volume (mL)	−2.0	0.0	Descriptive
Mean change in tumor volume (mL)	−12.5	+1.6	

## Discussion

In this retrospective cohort of patients with LGG, serial contrast-enhanced MRI demonstrated predominantly stable disease during follow-up, although radiological progression was more frequent among patients who underwent subtotal resection than among those managed with observation. Progression occurred in 18 (30.5%) of patients following subtotal resection compared with four (12.5%) in the observation group; however, this difference did not reach statistical significance (two-sided Fisher’s exact test, p=0.073). Tumor volume trajectories were broadly consistent with these categorical findings, with a reduction in mean tumor volume following subtotal resection and minimal overall change among observed patients.

The higher observed proportion of radiological progression in the subtotal resection group should not be interpreted as evidence that surgery itself increased the risk of tumor progression. Treatment allocation was based on clinical considerations rather than randomization, and therefore the management groups may have differed in baseline disease characteristics. In particular, patients selected for surgical intervention may have had larger or more symptomatic tumors, different anatomical locations, or other clinical features associated with a higher underlying risk of progression. Baseline tumor size, symptom status, tumor location, histological grade, and molecular profile were not consistently available in the retrospective dataset and therefore could not be reliably incorporated into an adjusted analysis. Accordingly, the observed association between management strategy and progression should be considered descriptive and potentially subject to confounding by indication rather than interpreted as a causal effect of surgery. Furthermore, subtotal resection inherently leaves residual tumor tissue, which may provide a substrate for subsequent radiological growth. Thus, the observed difference may reflect a combination of residual tumor burden and baseline differences in tumor biology and clinical characteristics rather than a direct adverse effect of surgery [9,11].

In contrast, patients managed with observation may have represented a clinically selected population with smaller, asymptomatic, relatively indolent, or surgically less accessible lesions. The predominance of stable disease in this group should therefore be interpreted in the context of the clinical selection of patients for surveillance rather than as evidence that observation is superior to surgical management [6,11]. Importantly, the subtotal resection and observation groups were not directly equivalent treatment populations because management was determined clinically rather than randomly. Accordingly, the present study describes radiological disease behavior within different management pathways and should not be interpreted as a comparative evaluation of treatment efficacy or as evidence of a causal treatment effect. Throughout the manuscript, comparisons between management groups are therefore presented as observational associations, with appropriate consideration of potential confounding and selection bias.

These findings are broadly consistent with previous evidence emphasizing the importance of extent of resection in the long-term management of LGG [9]. At the same time, the substantial proportion of patients who remained radiologically stable after subtotal resection supports the role of structured postoperative surveillance in patients with residual disease. Because progression may occur despite an initially stable postoperative appearance, regular MRI follow-up remains important for identifying changes in residual tumor burden and informing subsequent management [6,12].

From a clinical perspective, patients with known residual tumors may benefit from individualized surveillance intervals based on tumor characteristics, clinical status, and available molecular information. Early identification of radiological progression may facilitate timely multidisciplinary reassessment and consideration of additional treatment, including further surgery, radiotherapy, chemotherapy, or molecularly targeted therapy when clinically and molecularly appropriate [13]. However, treatment decisions should not be based on radiological volume change alone and should incorporate molecular classification, neurological status, symptoms, and patient-specific factors.

This study was limited by its single-center retrospective design, non-randomized treatment allocation, small sample size, and potential selection and confounding bias. The observation group and progression events were small, and the difference in progression rates was not statistically significant (p=0.073). Molecular information was incomplete. IDH status was unavailable for patients, and 1p/19q status was unavailable for patients. Follow-up information was also incomplete or insufficiently standardized, with patients lacking adequate standardized follow-up information for detailed longitudinal assessment. Consequently, molecular characteristics could not be reliably incorporated into group comparability assessments or adjusted analyses.

Importantly, progression in this study was not classified using formal RANO 2.0 criteria. Because RANO 2.0 assessment was not applied consistently throughout the retrospective study period, radiological progression was defined using the study-specific volumetric criterion of an increase in the existing tumor volume compared with the baseline MRI measurement. This definition should therefore not be interpreted as equivalent to a formal RANO 2.0 progression assessment. Overall survival, malignant transformation, standardized MRI follow-up intervals, and comprehensive molecular profiling were not consistently available. These limitations restrict interpretation of the observed radiological trajectories and preclude causal conclusions regarding the relative effectiveness of different management strategies.

## Conclusions

In this MRI-based retrospective cohort of patients with LGG, most patients remained radiologically stable during the observed follow-up period. Different proportions of radiological progression were observed between the management groups, with progression more frequently observed among patients who underwent subtotal resection; however, the comparison was not statistically significant, and the non-randomized design and potential baseline differences between groups preclude attribution of these findings to the management strategy itself. Tumor volume trajectories were consistent with the observed patterns of radiological stability and progression. Overall, this study describes imaging outcomes observed within different clinical management pathways rather than establishing the superiority or efficacy of a specific surveillance or treatment strategy. The findings underscore the importance of individualized clinical assessment and continued MRI surveillance, particularly in patients with residual tumor, while prospective studies with standardized imaging, molecular characterization, and adjustment for baseline prognostic factors are needed to further evaluate long-term outcomes.

## References

[1] Louis DN, Perry A, Wesseling P (2021). The 2021 WHO classification of tumors of the central nervous system: a summary. Neuro Oncol.

[2] Malta TM, Sabedot TS, Morosini NS (2024). The epigenetic evolution of gliomas is determined by their IDH1 mutation status and treatment regimen. Cancer Res.

[3] Weller M, van den Bent M, Preusser M (2021). EANO guidelines on the diagnosis and treatment of diffuse gliomas of adulthood. Nat Rev Clin Oncol.

[4] Aghi MK, Nahed BV, Sloan AE, Ryken TC, Kalkanis SN, Olson JJ (2015). The role of surgery in the management of patients with diffuse low grade glioma: a systematic review and evidence-based clinical practice guideline. J Neurooncol.

[5] Xia L, Fang C, Chen G, Sun C (2018). Relationship between the extent of resection and the survival of patients with low-grade gliomas: a systematic review and meta-analysis. BMC Cancer.

[6] Wen PY, van den Bent M, Youssef G (2023). RANO 2.0: update to the Response Assessment in Neuro-Oncology criteria for high-and low-grade gliomas in adults. J Clin Oncol.

[7] Mellinghoff IK, van den Bent MJ, Blumenthal DT (2023). Vorasidenib in IDH1-or IDH2-mutant low-grade glioma. N Engl J Med.

[8] Ellingson BM, Sanvito F, Cloughesy TF (2024). A neuroradiologist’s guide to operationalizing the Response Assessment in Neuro-Oncology (RANO) criteria version 2.0 for gliomas in adults. AJNR Am J Neuroradiol.

[9] Elsheikh M, Bridgman E, Lavrador JP, Lammy S, Poon MT (2022). Association of extent of resection and functional outcomes in diffuse low-grade glioma: systematic review & meta-analysis. J Neurooncol.

[10] (2026). CPT® 70552, under diagnostic radiology (diagnostic imaging) procedures of the head and neck. https://www.aapc.com/codes/cpt-codes/70552?srsltid=AU7gw4UGRhia3AmVoMGtu1VsyZj9_mIV7mT85rvqWp9lDy5W-fdtbh5f.

[11] Deacu M, Popescu S, Docu Axelerad A (2022). Prognostic factors of low-grade gliomas in adults. Curr Oncol.

[12] Baumert BG, P M Jaspers J, Keil VC (2025). ESTRO-EANO guideline on target delineation and radiotherapy for IDH-mutant WHO CNS grade 2 and 3 diffuse glioma. Radiother Oncol.

[13] Zhao Y, Xu Z, Liu Y (2025). Analysis of epidemiology and nomogram construction for prediction and clinical decision-making in gliomas. Front Immunol.

